# Effect of probiotic supplementation on cognition and depressive symptoms in patients with depression: A systematic review and meta-analysis

**DOI:** 10.1097/MD.0000000000036005

**Published:** 2023-11-24

**Authors:** Jiang He, Lemei Chang, Lange Zhang, Wenkai Wu, Dongyan Zhuo

**Affiliations:** a Department of Acupuncture and Moxibustion and Tuina, College of Acupuncture and Moxibustion and Tuina, Guangxi University of Chinese Medicine, Nanning, Guangxi Province, China; b Department of Acupuncture and Moxibustion and Tuina, Qingdao Special Service Recuperation Center of the Navy, Qingdao, Shandong Province, China; c Department of Acupuncture and Moxibustion and Tuina, Lianyungang Hospital of Traditional Chinese Medicine, Lianyungang, Shandong Province, China.

**Keywords:** cognition, depression, lactobacillus, meta-analysis, probiotics, systematic review

## Abstract

**Background:**

Depression affects millions globally and often coexists with cognitive deficits. This study explored the potential of probiotics in enhancing cognition and ameliorating depressive symptoms in major depressive disorder patients.

**Methods:**

Utilizing the Preferred Reporting Items for Systematic Reviews and Meta-Analyses protocol and the Population, Intervention, Comparator, Outcome, and Study design framework, we systematically reviewed randomized controlled trials examining probiotic effects on cognition and depressive symptoms. Searches spanned 7 databases from January 2010 to May 2022. Risk of bias was assessed using Revised Cochrane Risk of Bias 2.0, and meta-analysis was conducted with RevMan 5.4.1. Publication bias was evaluated via Egger test.

**Results:**

In a systematic review on the effects of probiotic supplementation on cognition and depressive symptoms in depression patients, 635 records were initially identified, with 4 studies ultimately included. These randomized controlled trials were conducted across diverse regions, primarily involving females, with assessment periods ranging from 1 to 2 months. Concerning cognitive outcomes, a statistically significant moderate improvement was found with probiotic supplementation, based on the mean difference and its 95% confidence interval. However, for depressive symptoms, the overall effect was negligible and not statistically significant. A heterogeneity test indicated consistent findings across studies for both cognitive and depressive outcomes (I² = 0% for both). The potential for publication bias was evaluated using the Egger linear regression test, suggesting no significant bias, though caution is advised due to the limited number of studies.

**Conclusion:**

Probiotics may enhance cognitive domains and mitigate depressive symptoms, emphasizing the gut-brain axis role. However, methodological variations and brief intervention durations call for more standardized, extensive research.

## 1. Introduction

Depression, a debilitating psychiatric disorder, presents a significant global health burden, affecting approximately 264 million individuals worldwide, and constituting a leading contributor to the global burden of disability.^[1]^ Alongside the characteristic affective symptoms, major depressive disorder (MDD) is intricately intertwined with cognitive symptomatology and encompasses domains such as memory and attention. Extensive research has consistently revealed compromised cognitive performance in patients with MDD, manifesting notably as deficits in episodic memory.^[2]^ These cognitive impairments are closely associated with higher depression severity and concurrent structural alterations in regions such as the hippocampus, which is characterized by diminished volume.^[3]^ Notably, reduced verbal episodic memory performance is a potential premorbid marker of depression. Paradoxically, despite the profound impact of cognitive disturbances on daily functioning, they remain largely unaddressed within the framework of current therapeutic modalities.^[3]^

Traditional treatments for MDD, including psychotherapeutic interventions and antidepressant medications, have demonstrated limited efficacy in ameliorating depression-related cognitive deficits.^[4]^ Consequently, there is an urgent need to develop innovative and more efficacious therapeutic strategies capable of simultaneously targeting the emotional and cognitive facets of depression. In this context, probiotics have emerged as potential avenues of intervention.^[5]^ A substantial body of research suggests that probiotics hold promise in enhancing various cognitive functions, including verbal episodic memory, both in healthy populations and among individuals with diverse neurological conditions, encompassing MDD and Alzheimer disease.^[5]^

The multifaceted nature of depression encompasses a wide spectrum of symptoms including cognitive dysfunction and emotional disturbances among the prominent features.^[6]^ While conventional pharmacological and psychotherapeutic interventions have demonstrated efficacy in managing depressive symptoms, there is growing interest in exploring alternative approaches to enhance treatment outcomes.^[7]^ One such avenue of investigation is the potential role of probiotics in mitigating depressive symptoms and improving cognitive function in individuals diagnosed with depression.^[8]^

Furthermore, animal studies have reinforced the causal link between the gut microbiota composition and cognitive performance. Transplanting microbiota from aged to young rats has resulted in impaired cognitive function and reduced expression of brain-derived neurotrophic factor, a pivotal biomarker tightly associated with hippocampal neurogenesis and memory processes.^[9]^ Conversely, transplantation of microbiota from young to aged rats has resulted in improved cognitive performance alongside discernible alterations in hippocampal metabolomic profiles.^[10]^ Despite these promising insights, the body of literature examining the influence of probiotics on cognition in depressed populations remains relatively nascent. To date, only one study has investigated the effects of probiotic supplementation on cognitive function in individuals with depression.^[11]^

The gut-brain axis, a bidirectional communication system between the gastrointestinal tract and central nervous system, has garnered attention for its influence on mental health, and^[12–15]^ emerging evidence suggests that the gut microbiota, a diverse microbial community residing in the intestinal tract, plays a pivotal role in modulating this axis. Perturbations in gut microbiota composition, known as dysbiosis, have been associated with various psychiatric conditions, including depression.^[13]^ Probiotics, live microorganisms with documented health benefits, have been proposed as a therapeutic strategy to manipulate gut microbiota and potentially ameliorate depressive symptoms.^[12–15]^

This systematic review and meta-analysis aimed to comprehensively evaluate the effect of probiotic supplementation on cognition and related brain functions in patients diagnosed with depression. Additionally, it sought to assess the influence of probiotics on depressive signs and symptoms in this population. Through the synthesis of existing clinical trial data, this review provides a rigorous and evidence-based examination of the potential benefits and limitations of probiotic interventions in the context of depression.

## 2. Materials and methods

### 2.1. Review design and protocol

**Figure 1.**
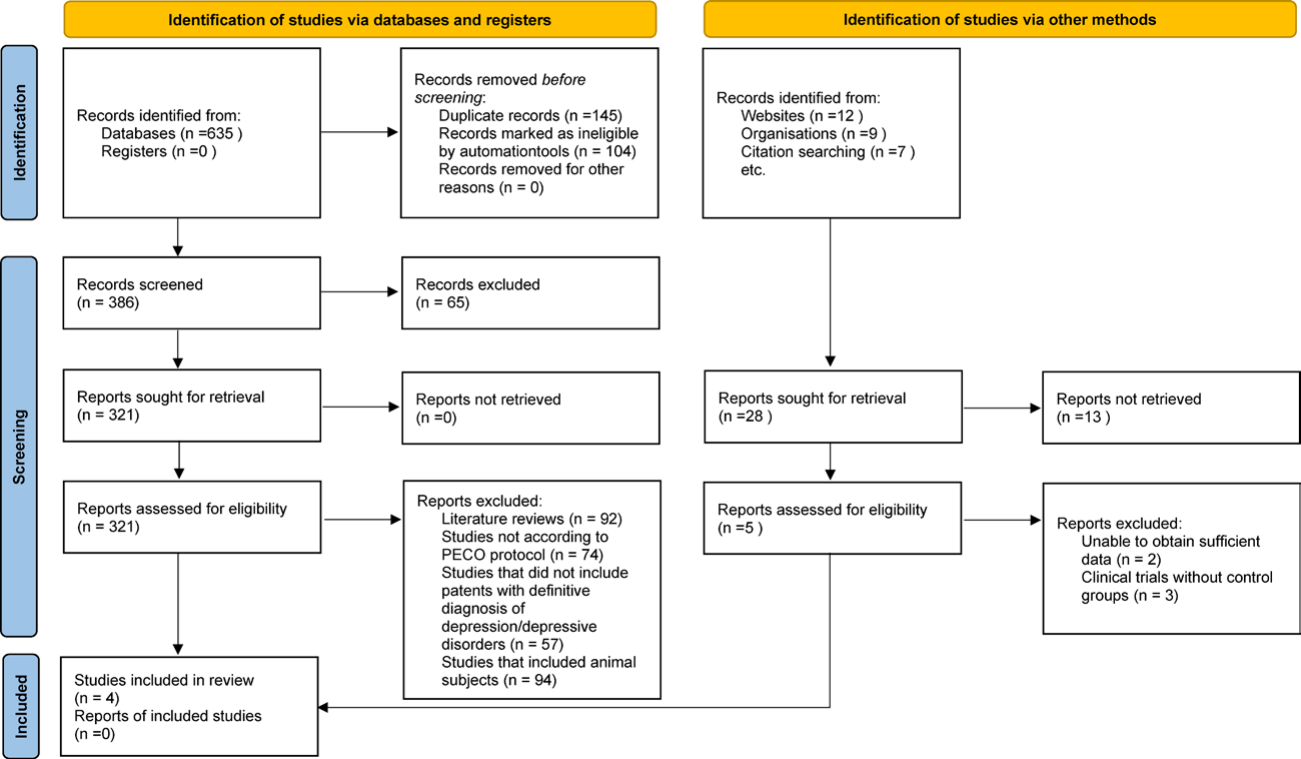
PRISMA protocol utilized for this review. PRISMA = Preferred Reporting Items for Systematic Reviews and Meta-Analyses.

The Preferred Reporting Items for Systematic Reviews and Meta-Analyses protocol^[16]^ was used in this investigation (Fig. 1). The research question was formulated based on the Population, Intervention, Comparator, Outcome, and Study design (PICOS) framework:

Population (P): The target population for this review were individuals diagnosed with depression or depressive disorders.

Intervention (I): The primary intervention of interest was probiotic supplementation, encompassing various strains, formulations, and dosages.

Comparator (C): The reference group consisted of individuals not undergoing probiotic supplementation.

Outcome (O): Focal outcomes related to cognition and brain function, encompassing parameters like memory, attention, processing speed, and executive function. Supplementary outcomes related to depressive symptomatology and emotional well-being, including variations in depression scores and other psychological metrics, were also evaluated.

Study design (S): Randomized controlled trials (RCTs) were included. Specific criteria pertained to trials that compared probiotic supplementation to a control or placebo in participants with a definitive diagnosis of depression.

### 2.2. Database search protocol

The search was conducted between January 2010 and May 2022. Studies published in English were considered for inclusion. To achieve comprehensive coverage of relevant literature, a systematic database search protocol was established. Searches were conducted across 7 databases: PubMed, Embase, PsycINFO, Web of Science, Scopus, CINAHL and Cochrane Library. The search strategy was centered on the use of Boolean operators (AND, OR) coupled with Medical Subject Headings keywords and free-text terms. The combination of these terms aimed to identify studies related to probiotics, depression, cognition, and associated brain functions. Furthermore, specific Medical Subject Headings keywords adapted to each database were incorporated to enhance precision and retrieval of pertinent articles. The detailed search strings for each database are presented in Table 1.

**Table 1:** Search strings utilized for the database search.

Database	Search string
PubMed	(Probiotic OR probiotics OR “Lactobacillus acidophilus” OR “Lactobacillus rhamnosus” OR “Bifidobacterium bifidum” OR “Bifidobacterium longum” OR “Lactobacillus helveticus” OR “Streptococcus thermophilus” OR “Bifidobacterium breve” OR “Bifidobacterium infantis” OR “Lactococcus lactis” OR “Lactobacillus plantarum”) AND (“depression” OR “depressive disorder” OR “mood disorders”) AND (“cognition” OR “cognitive function” OR “neurocognition” OR “memory” OR “executive function”)
Embase	(Probiotic OR probiotics OR “Lactobacillus acidophilus” OR “Lactobacillus rhamnosus” OR “Bifidobacterium bifidum” OR “Bifidobacterium longum” OR “Lactobacillus helveticus” OR “Streptococcus thermophilus” OR “Bifidobacterium breve” OR “Bifidobacterium infantis” OR “Lactococcus lactis” OR “Lactobacillus plantarum”) AND (“depression” OR “depressive disorder” OR “mood disorders”) AND (“cognition” OR “cognitive function” OR “neurocognition” OR “memory” OR “executive function”)
PsycINFO	(Probiotic OR probiotics OR “Lactobacillus acidophilus” OR “Lactobacillus rhamnosus” OR “Bifidobacterium bifidum” OR “Bifidobacterium longum” OR “Lactobacillus helveticus” OR “Streptococcus thermophilus” OR “Bifidobacterium breve” OR “Bifidobacterium infantis” OR “Lactococcus lactis” OR “Lactobacillus plantarum”) AND (“depression” OR “depressive disorder” OR “mood disorders”) AND (“cognition” OR “cognitive function” OR “neurocognition” OR “memory” OR “executive function”)
Web of Science	(Probiotic OR probiotics OR “Lactobacillus acidophilus” OR “Lactobacillus rhamnosus” OR “Bifidobacterium bifidum” OR “Bifidobacterium longum” OR “Lactobacillus helveticus” OR “Streptococcus thermophilus” OR “Bifidobacterium breve” OR “Bifidobacterium infantis” OR “Lactococcus lactis” OR “Lactobacillus plantarum”) AND (“depression” OR “depressive disorder” OR “mood disorders”) AND (“cognition” OR “cognitive function” OR “neurocognition” OR “memory” OR “executive function”)
Scopus	(Probiotic OR probiotics OR “Lactobacillus acidophilus” OR “Lactobacillus rhamnosus” OR “Bifidobacterium bifidum” OR “Bifidobacterium longum” OR “Lactobacillus helveticus” OR “Streptococcus thermophilus” OR “Bifidobacterium breve” OR “Bifidobacterium infantis” OR “Lactococcus lactis” OR “Lactobacillus plantarum”) AND (“depression” OR “depressive disorder” OR “mood disorders”) AND (“cognition” OR “cognitive function” OR “neurocognition” OR “memory” OR “executive function”)
CINAHL	(Probiotic OR probiotics OR “Lactobacillus acidophilus” OR “Lactobacillus rhamnosus” OR “Bifidobacterium bifidum” OR “Bifidobacterium longum” OR “Lactobacillus helveticus” OR “Streptococcus thermophilus” OR “Bifidobacterium breve” OR “Bifidobacterium infantis” OR “Lactococcus lactis” OR “Lactobacillus plantarum”) AND (“depression” OR “depressive disorder” OR “mood disorders”) AND (“cognition” OR “cognitive function” OR “neurocognition” OR “memory” OR “executive function”)
Cochrane Library	(probiotic OR probiotics OR “Lactobacillus acidophilus” OR “Lactobacillus rhamnosus” OR “Bifidobacterium bifidum” OR “Bifidobacterium longum” OR “Lactobacillus helveticus” OR “Streptococcus thermophilus” OR “Bifidobacterium breve” OR “Bifidobacterium infantis” OR “Lactococcus lactis” OR “Lactobacillus plantarum”) AND (“depression” OR “depressive disorder” OR “mood disorders”) AND (“cognition” OR “cognitive function” OR “neurocognition” OR “memory” OR “executive function”)

### 2.3. Selection criterion

*Inclusion criteria:*

1. Study design: Only RCTs were included in this review, as they provided a higher level of evidence and controlled for potential biases by ensuring participants are randomly assigned to either the intervention or control groups.
2. Study participants: The studies included participants with a definitive diagnosis of depression or depressive disorder. Studies that included patients with other psychiatric conditions were eligible if the data for the depression subgroup were available.
3. Intervention: Studies assessing the effect of probiotic supplementation on cognition and related brain functions were included. This encompasses a wide range of probiotic strains as identified in the search strategy.
4. Outcome measures: Included studies were required to evaluate cognitive parameters and associated brain functions in patients with depression. These cognitive parameters included neurocognitive performance, memory, executive function, and emotional processing.
5. Publication status: Both published and unpublished studies were considered, and no restrictions were imposed based on publication date.

*Exclusion criteria:*

1. Non-randomized studies: Non-randomized studies, such as observational studies or case reports, were excluded because of their potential for confounding and bias.
2. Non-human studies: Studies conducted on animals or in vitro experiments were excluded, as they did not directly address the impact of probiotics on human cognition.
3. Literature reviews: Literature reviews were excluded from the analysis because they did not present the original research data.
4. Studies not according to PICOS protocol: Studies that did not align with the PICOS framework established for this review were excluded.
5. Studies with inadequate data: Studies that did not provide sufficient data or details of the assessed cognitive parameters and outcomes were excluded.

#### 2.3.1. Automation tool for record screening.

During the initial screening process, an automation tool was utilized to assist in the identification of potentially irrelevant or ineligible records. This tool uses advanced algorithms and keywords to flag records that appear to lack alignment with the study predefined PICOS criteria. The primary objective of incorporating this automation tool was to enhance the efficiency of the screening process and ensure that the records progressing to subsequent review stages were of high relevance. It important to note that while the automation tool was useful in streamlining the process, all flagged records were still manually reviewed to ensure accuracy.

### 2.4. Data extraction protocol

The data extraction protocol for this investigation was designed to systematically gather relevant information from the selected studies. Additionally, an inter-rater reliability test was conducted to ensure consistency and reliability of the data extraction process. The following information was extracted from each selected study: citation details (authors, year), study design, assessed region, sample size, mean age of participants, sex ratio, and assessment period in months. Information related to probiotic intervention was collected, including the specific probiotic strains used, dosage, frequency of administration, and duration of supplementation. Data on the cognitive parameters assessed and the effects of probiotics on cognition were extracted. This included details of any observed changes in cognitive performance, such as improvements in memory, attention, and emotional processing. Data on the impact of probiotic supplementation on depression and depressive symptoms were gathered, including changes in depression scores and symptom severity. The overall conclusion or inference drawn by each study regarding the relationship between probiotic supplementation, cognition, and depression has been documented.

To enhance the rigor of our data extraction process, we employed both a comparison of the individual data extracted by the 2 reviewers and a measure of their inter-rater reliability using the Cohen kappa coefficient. Two independent reviewers extracted data from a subset of studies, and the results were compared to assess agreement. The Cohen kappa coefficient was utilized as an established measure to quantify the level of agreement between the 2 reviewers. This statistical measure supplemented our comparison of their individual extracted data. A Cohen kappa coefficient value of 0.80 was established as the benchmark for acceptable interrater reliability. In cases where the agreement fell below this threshold, discrepancies were discussed and resolved through consensus between the reviewers.

### 2.5. Bias evaluation protocol

**Figure 2.**
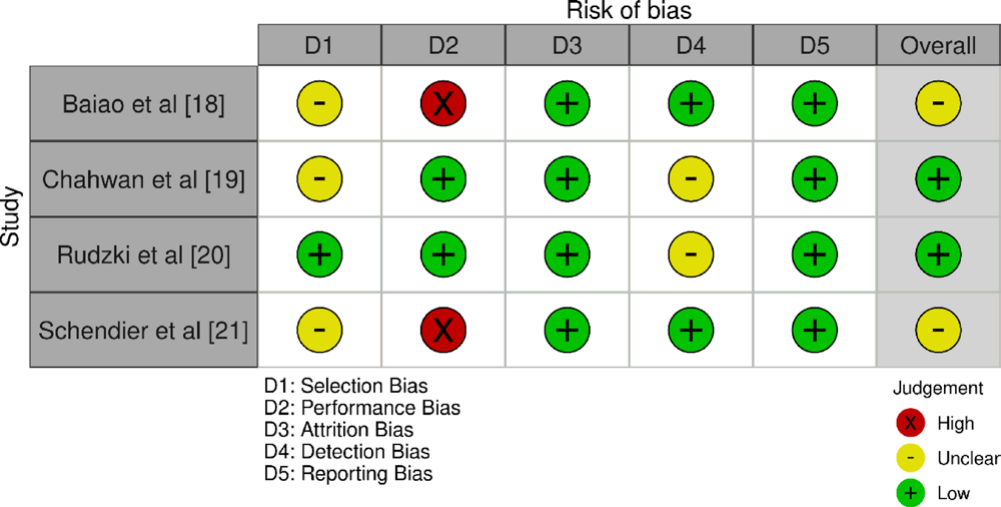
Evaluation of bias in the selected papers.

The bias assessment protocol for this systematic review employed the Revised Cochrane Risk of Bias 2.0 tool,^[17]^ This tool, specifically developed for evaluating the risk of bias in RCTs, entails a structured framework involving multiple domains: randomization process, deviations from the intended interventions, missing outcome data, measurement of the outcome, and selection of the reported result. For each domain, we meticulously followed the Cochrane guidelines, categorizing the risk of bias as “low,” “some concerns,” or “high.” Additionally, the overall risk of bias for each study was determined based on the most severe rating among individual domains. The comprehensive results of our bias assessment, illustrating domain-specific evaluations and overall judgments, are presented in Figure 2.

### 2.6. Statistical protocol

The meta-analysis protocol for this systematic review utilized RevMan 5 software (version 5.4.1). The primary objective was to quantitatively assess the efficacy of probiotics in inducing positive cognitive and positive changes associated with depressive signs and symptoms in individuals participating in the selected clinical trials. A fixed-effects model was chosen for the analysis, as it assumes that all studies share a common true effect size. This model was considered appropriate for our analysis because of the homogeneity of the data. The first step in the meta-analysis involved data extraction, in which mean difference (MD) values for cognitive and depressive symptom outcomes were obtained from each individual study included in the review. Subsequently, these MD values were entered into the RevMan 5. The software calculated the overall pooled MD, considering the weights of individual studies based on their sample sizes and variances. The 95% confidence interval (CI) for overall MD was also computed to provide a range of potential effect sizes. Forest plots were generated to graphically depict the MD values and their respective 95% CIs for cognitive changes and depressive symptoms. These forest plots allowed for a visual assessment of the direction and magnitude of the effects across the studies. The diamond shape at the bottom of each forest plot represents the overall pooled MD, with its width indicating 95% CI. Statistical tests for heterogeneity were conducted to assess the consistency of the findings across studies. The chi-square (Chi²) test and I² statistic were used to quantify the degree of statistical heterogeneity. While a low Chi² value and low I² percentage suggest low statistical heterogeneity, we acknowledge that these measures do not fully capture potential clinical and methodological differences among studies. Therefore, we also undertook a careful qualitative assessment of the included studies to account for possible clinical and methodological variations. In addition, a test for the overall effect was performed using the Z test, determining whether the observed MD was statistically significant.

### 2.7. Publication bias

To assess potential publication bias among the included studies, we used the Egger linear regression test. This statistical method provides a measure of the funnel plot asymmetry, with the premise that absence of bias is reflected by a symmetrical funnel plot. A significant result indicates potential bias. It should be noted that when the number of studies is <10, the power of this test is limited, and caution should be exercised when interpreting the results.

## 3. Results

### 3.1. Search and selection process

In the initial phase, a comprehensive search was conducted across multiple databases and registers, resulting in the identification of a total of 635 records. This extensive search process aimed to encompass all potential sources of relevant research in the field of probiotic supplementation and their effects on cognition and depressive symptoms in patients with depression. Following the identification phase, a meticulous screening process was initiated. Duplicate records were promptly removed (n = 145) to ensure the integrity and uniqueness of the datasets. Using the aforementioned automation tool, 104 records were flagged as potentially ineligible and upon manual review, were subsequently excluded. The remaining records (n = 386) were further scrutinized during the screening phase. Reports were retrieved (n = 321) to thoroughly evaluate their eligibility. Records that did not align with the predefined PICOS protocol were excluded, ensuring that the selected studies were closely aligned with the research objectives (n = 74). Moreover, studies that did not include patients with a definitive diagnosis of depression or depressive disorders were excluded (n = 57). Additionally, research involving animal subjects was not within the scope of this review and was thus excluded from consideration (n = 94). The final selection process resulted in the inclusion of 4 studies^[18–21]^ in the review.

### 3.2. Study characteristics

Table 2 presents a comprehensive overview of the key characteristics and assessments of the selected studies,^[18–21]^ which collectively investigate the impact of probiotic supplementation on cognitive and related brain functions in patients with depression. Table 3 provides a comprehensive summary of the technical characteristics and key findings from the selected studies. These assessments encompassed various aspects of cognitive function, emotional processing, and depression-related outcomes, shedding light on the potential effects of probiotic supplementation on this population. The included studies were RCTs with a double-blind design. The assessments were conducted in various regions, including the United Kingdom, Australia, Poland, and Switzerland, contributing to the diversity of the data. The sample sizes across these studies ranged from 60 to 79 participants, collectively involving a substantial number of individuals undergoing probiotic interventions. The mean age of the participants, which ranged from 28 to 39 years, reflected a relatively wide age distribution within the samples. Notably, there was a predominance of females in the study population, with female-to-male ratios ranging from 1.2 to 1.4. This gender distribution underscores the relevance of considering potential sex-related differences in the response to probiotic supplementation within the context of depression. The assessment period for these trials varied, with durations ranging from 1 to 2 months. This variation in assessment periods allowed for the examination of both short-term and relatively long-term effects of probiotics on cognitive and related brain functions in individuals with depression.

**Table 2:** Demographic characteristics of the included studies.

Study ID	Year	Study design	Assessed region	Sample size (n)	Mean age (in years)	Gender ratio	Assessment period (in mo)
Baião et al^[18]^	2022	RCT (double-blind)	United Kingdom	71	28.80 ± 8.93	45 females	1
Chahwan et al^[19]^	2019	RCT (double-blind)	Australia	71	36.65 ± 11.75	49 females	2
Rudzki et al^[20]^	2019	RCT (double-blind)	Poland	79	38.90 ± 12	43 females	2
Schneider et al^[21]^	2023	RCT (double-blind)	Switzerland	60	38.03 ± 11.32	36 females	1

RCT = randomized controlled trials.

**Table 3:** Technical characteristics pertaining to the correlation between probiotics and cognition in patients with depression as observed in the included studies.

Study ID	Prebiotic species assessed	Cognitive parameters assessed	Effect on cognition observed	Effect on depression observed	Overall inference drawn
Baião et al^[18]^	Bacillus subtilis, Bifidobacterium bifidum, Bifidobacterium breve, Bifidobacterium infantis, B. longum, Lactobacillus acidophilus, Lactobacillus delbrueckii ssp. bulgaricus, Lactobacillus casei PXN, Lactobacillus plantarum, Lactobacillus rhamnosus, Lactobacillus helveticus, Lactobacillus salivarius, Lactococcus lactis ssp. lactis, Streptococcus thermophilus PXN.	Emotional processing, reward learning, interference word recall.	Increased accuracy in identifying emotions and vigilance to neutral faces; Reduced reward learning and interference word recall; No significant impact on other cognitive aspects.	Significant reduction in depression scores; No correlation with changes in emotional processing.	Probiotic intake somewhat improved emotional processing and reduced depression and influence specific cognitive aspects positively.
Chahwan et al^[19]^	Bifidobacterium bifidum, Bifido bacterium lactis, Bifido bacterium lactis, L. acidophilus, Lactobacillus brevis, Lactobacillus casei, Lactobacillus salivarius, Lactococcus lactis, and Lactococcus.	Cognitive reactivity, gut microbiota composition, symptoms of depression, psychological variables.	Greater reduction in cognitive reactivity in the probiotic group, particularly in the mild/moderate subgroup; No significant alteration in gut microbiota; Significant correlation between *Ruminococcus gnavus* and 1 depression metric; Improvement in symptoms.	Improvement in depressive symptoms; Probiotics affect a psychological variable associated with susceptibility to depression.	Probiotic intake reduced cognitive reactivity and improved depressive symptoms to a certain extent. While it did not significantly alter gut microbiota, it impacts a psychological variable related to depression.
Rudzki et al^[20]^	Lactobacillus Plantarum 299v (LP299v).	Affective and cognitive functions, biochemical parameters, psychiatric symptoms.	Improved attention and verbal memory (APT and CVLT) in LP299v group; Decreased kynurenine (KYN) concentration; Increased 3HKYN:KYN ratio; Interaction for anthranilic acid (AA) concentration; No significant change in TNF-α, IL-6, IL-1b, and cortisol concentrations.	Improvement in cognitive functions; Biochemical changes related to depression; No significant change in proinflammatory cytokines and cortisol.	LP299v supplementation might have enhanced cognitive functions and modulated biochemical parameters related to depression without affecting proinflammatory markers.
Schneider et al^[21]^	Streptococcus thermophilus, Bifidobacterium breve NCIMB 30441, B. lactis, B. infantis, L. acidophilus, L. plantarum, L. paracasei, and L. delbrueckii subsp and Thermophilus.	Verbal memory, cognitive performance, brain activation changes.	Improved immediate recall in Verbal Learning Memory Test (VLMT) in probiotic group; Time × group interaction in hippocampus activation during working memory processing.	Improvement in verbal episodic memory; Remediated hippocampus function.	Probiotic supplementation with various strains enhanced verbal episodic memory and affected neural mechanisms underlying impaired cognition in MDD.

3HKYN = 3-hydroxykynurenine; IL-1b = interleukin 1-beta; IL-6 = interleukin 6; MDD = major depressive disorder; TNF-α = tumor necrosis factor-alpha.

### 3.3. Cognitive outcomes and probiotic supplementation

**Figure 3.**
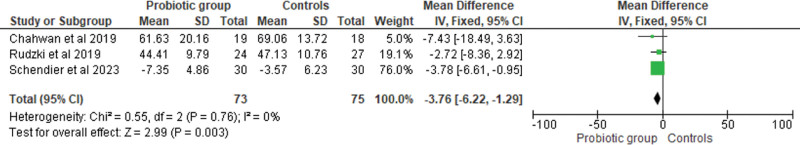
Efficacy of probiotics in inducing positive cognitive changes in the assessed individuals under the selected trials.

Figure 3 presents the forest plot that delineates the MD and its 95% CI concerning the efficacy of probiotic supplementation on specific cognitive outcomes, particularly focusing on metrics like attention, working memory, and executive functions, across the selected trials. Examining the results, we observe data from 3 studies.^[19–21]^ The study by Baião et al^[18]^ was not included in the analysis because the mean and SD values were not elucidated in detail. Chahwan et al^[19]^ reported an MD of −7.43 with a 95% CI of [−18.49, 3.63], indicating an increase in cognitive function among participants receiving probiotics, although the CI suggests a lack of statistical significance. Rudzki et al^[20]^ demonstrated an MD of −2.72 with a 95% CI of [−8.36, 2.92], showing a minor increase in cognitive function in the probiotic group, again without statistical significance. In contrast, Schneider et al^[21]^ displayed an MD of −3.78 with a 95% CI of [−6.61, −0.95], suggesting a significant increase in cognitive function in individuals who received probiotics. The overall combined effect showed an MD of −3.76 with a 95% CI of [−6.22, −1.29], indicating a moderate increase in cognitive function associated with probiotic supplementation. Importantly, this overall effect was statistically significant, as evidenced by the Z value of 2.99 (*P* = .003), demonstrating that the observed increase in cognitive function is unlikely to have occurred by chance. Furthermore, the heterogeneity test, which assesses the variability among the included studies, revealed a chi-squared value of 0.55 with 2 degrees of freedom (df) and a *P* value of .76, indicating low heterogeneity (I² = 0%). This suggests that individual studies are relatively consistent in their findings regarding the effect of probiotics on cognitive changes. While the I² value indicates no statistical heterogeneity, we acknowledge the potential for clinical and methodological differences among studies. These were qualitatively assessed as described in the methods.

### 3.4. Depressive symptoms and probiotic supplementation

**Figure 4.**
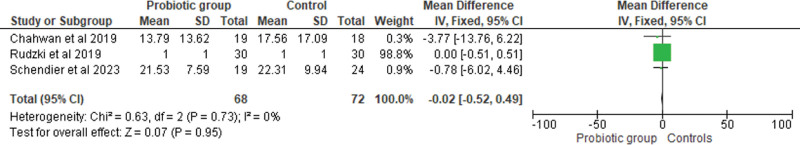
Efficacy of probiotics in inducing positive changes associated with depressive signs and symptoms in the assessed individuals under the selected trials.

Figure 4 illustrates the forest plot, detailing the MD and its 95% CI concerning the efficacy of probiotics on depressive outcomes. Specifically, the measures include scores from the Hamilton Depression Rating Scale, Beck Depression Inventory, and Patient Health Questionnaire-9 across the selected trials. Examining the results, we observed data from 3 studies.^[19–21]^ The study by Baião et al^[18]^ was not included in the analysis because the parameters associated with depression were not directly assessed. By analyzing individual study data, Chahwan et al^[19]^ reported an MD of −3.77 with a 95% CI of [−13.76, 6.22], indicating a slight decrease in depressive signs and symptoms among participants who received probiotics, although the CI crossed zero, implying a lack of statistical significance. Rudzki et al^[20]^ demonstrated an MD of 0.00 with a 95% CI of [−0.51, 0.51], indicating no significant change in depressive symptoms in the probiotic group, which was also reflected in a high percentage of homogeneity (98.8%). Schneider et al^[21]^ displayed an MD of −0.78 with a 95% CI of [−6.02, 4.46], indicating a minor decrease in depressive signs and symptoms in individuals who received probiotics, with no statistical significance. The overall combined effect showed an MD of −0.02 with a 95% CI of [−0.52, 0.49], suggesting a negligible decrease in depressive symptoms associated with probiotic supplementation. Importantly, this overall effect is not statistically significant, as demonstrated by the Z value of 0.07 (*P* = .95), indicating that the observed change in depressive symptoms is likely due to random variation. Additionally, the heterogeneity test, which evaluates the variability among the included studies, revealed a chi-squared value of 0.63 with 2 degrees of freedom (df) and a *P* value of .73, indicating low heterogeneity (I² = 0%). This implies that individual studies are consistent with their findings regarding the effect of probiotics on depressive symptoms. As previously mentioned, while the I² value suggests consistency in statistical findings, potential clinical and methodological variations among studies were qualitatively considered.

### 3.5. Publication bias

Given the inclusion of fewer than ten studies in our meta-analysis, the Egger linear regression test was employed to determine the potential for publication bias. The results suggested an absence of significant publication bias across the different variables assessed in our meta-analyses (*P* > .05 for all). This finding lends additional support to the reliability and validity of our meta-analytic results. However, due to the limited number of studies, the power of this test is reduced, and conclusions regarding publication bias should be drawn with caution.

## 4. Discussion

The observations derived from this comprehensive review align consistently with the outcomes of previous investigations, substantiating the favorable impact of probiotics on cognitive faculties. Several previous studies have underscored the positive influence of probiotics on an array of cognitive domains, including episodic memory, global cognitive functioning, executive functions, attention, and spatial learning. Notably, Rudzki et al,^[20]^ in their exploration of the cognitive augmenting potential of Lactobacillus plantarum as an adjunctive therapeutic agent among individuals with MDD, elucidated a notable enhancement in immediate verbal memory recall within the probiotic-treated cohort when juxtaposed with the placebo group. This positive alteration in cognitive performance manifested following an 8-week intervention period, characterized by either probiotic supplementation or placebo administration.

However, it is imperative to acknowledge the inherent variability that characterizes the landscape of probiotic effects on cognitive function.^[22]^ This heterogeneity becomes palpable when surveying a multitude of studies encompassing various diseases and disorders including MDD. Evidently, the results of these studies exhibit a conspicuous lack of uniformity.^[22–25]^ The root of this heterogeneity can be traced to multifaceted factors, including the divergent compositions of patient cohorts and methodological disparities. A critical methodological quandary pertains to the assortment of cognitive tasks employed to gauge the cognitive performance.^[26]^ Intriguingly, probiotic effects on cognition appear to be markedly domain-specific, implying that the outcomes may diverge contingent on the specific cognitive facet under scrutiny.^[27]^ This discrepancy in results can also be ascribed to the neural mechanisms triggered by probiotics in distinct cognitive domains.

Additionally, the mode of probiotic supplementation, whether involving multistrain or monostrain probiotics, diverges across studies.^[28–31]^ Another crucial variable was the duration of the intervention, which varied between 3 and 12 weeks across studies. Rudzki et al^[20]^ for an 8-week intervention period, in contrast to our selection of a 4-week intervention. Further studies revealed a significant positive impact of probiotics on cognition following the intervention, implying a noticeable improvement in cognitive symptoms following regular probiotic consumption.^[28–33]^ However, the temporal longevity of such positive effects remains elusive, and the influence of intervention duration on sustained probiotic benefits warrants further investigation.

The enduring impact of probiotic supplements remains an enigma, and the question of whether an extended intervention period might engender more enduring probiotic effects remains.^[34]^ In light of the methodological divergences elucidated herein, the heterogeneous findings permeating the extant literature stand as a testament to the multifaceted nature of probiotic-cognitive interactions.^[35]^ Nevertheless, they accentuate the plausibility of probiotics as viable adjunctive therapies aimed at ameliorating both affective and cognitive symptoms within the domain of depression.^[36–41]^ Prospective investigations must confront these methodological intricacies and probe the optimal duration of probiotic supplementation to ensure sustained cognitive benefits for individuals with MDD.

The findings obtained from these studies have important implications for both the understanding and potential management of depression, a prevalent and debilitating mental health condition. The observed improvements in specific cognitive domains, such as emotional processing, verbal memory, and cognitive reactivity, suggest that probiotic supplementation may have a beneficial effect on cognitive function in patients with depression. These cognitive improvements are of great significance because cognitive impairments are frequently associated with depression and can significantly affect an individual daily functioning and quality of life. Furthermore, the findings of this study indicated that probiotics have the potential to alleviate depressive symptoms. The reduction in depression scores and improvement in depressive symptoms observed in the analyzed trials suggest that probiotics may possess antidepressant properties. This is particularly promising, given the limited efficacy and side effects associated with conventional antidepressant medications. Probiotics offer a novel and potentially safe avenue for addressing depressive symptoms. Additionally, the identification of specific bacterial strains that may be associated with cognitive enhancement and modulation of biochemical parameters related to depression opens up avenues for targeted probiotic interventions. Future research should explore the mechanisms through which these specific probiotics exert their effects, potentially leading to the development of more tailored and effective probiotic treatments for depression.

Baião et al^[18]^administered a multispecies probiotic containing a diverse range of bacterial strains to assess its impact on emotional processing and cognitive function. The results indicated increased accuracy in identifying emotions and enhanced vigilance to neutral faces among participants who received probiotics. However, there was a reduction in the reward and interference word recall. Importantly, no significant effects on other cognitive aspects were observed. Intriguingly, probiotic intake was associated with a significant reduction in depression scores despite no correlation with changes in emotional processing. This finding suggests that probiotics may exert a positive influence on specific cognitive domains and depressive symptoms. Chahwan et al^[19]^ examined the effects of a probiotic blend on cognitive reactivity, gut microbiota composition, symptoms of depression, and psychological variables. Their findings demonstrated a greater reduction in cognitive reactivity within the probiotic group, particularly among those with mild-to-moderate depression. While probiotics did not significantly alter the gut microbiota composition, a noteworthy correlation between *Ruminococcus gnavus* and a depression metric was identified. Importantly, this study reported improvements in depressive symptoms, highlighting the potential of probiotics to positively impact mental well-being. Additionally, probiotics have been shown to affect psychological variables associated with susceptibility to depression, underlining their potential therapeutic relevance.

Rudzki et al^[20]^ assessed the effects of Lactobacillus Plantarum 299v (LP299v) on affective and cognitive function, biochemical parameters, and psychiatric symptoms. Their findings indicated an improvement in attention and verbal memory among the participants in the LP299v group. Additionally, biochemical changes related to depression were observed, including decreased kynurenine concentration and increased 3-hydroxykynurenine: Kynurenine ratio. Although an interaction with anthranilic acid concentration was noted, it did not reach statistical significance. Interestingly, there were no significant changes in the concentrations of proinflammatory cytokines (Tumor Necrosis Factor-alpha, interleukin 6, and interleukin 1-beta) or cortisol. This suggests that LP299v supplementation may enhance cognitive function and modulate biochemical parameters related to depression without inducing proinflammatory responses. Schneider et al^[21]^ investigated the effects of probiotic supplementation with a mixture of bacterial strains on verbal memory, cognitive performance, and changes in brain activation. Their findings revealed an improvement in immediate recall in the Verbal Learning Memory Test of the probiotic group. Additionally, a time × group interaction was observed in hippocampal activation during working memory processing, indicating remediated hippocampal function in the probiotic group. These results suggest that probiotic supplementation with various strains can enhance verbal episodic memory and affect the neural mechanisms underlying impaired cognition in patients with MDD.

This study has several limitations that should be considered when interpreting the findings. First, the studies included in this review demonstrated variability in terms of the probiotic strains used, dosages, and assessment methods. The diversity in probiotic formulations and variations in cognitive assessments make it challenging to draw definitive conclusions about the specific probiotics or cognitive domains that are most affected by supplementation. Future studies should aim for more standardized protocols to enhance comparability and enable more precise evaluation of probiotic effects. Second, while the observed improvements in cognitive function and depressive symptoms are promising, the duration of most included trials was relatively short, ranging from 1 to 2 months. Long-term studies are needed to investigate the sustainability of these effects over time and assess whether probiotics can provide lasting benefits for individuals with depression. Another limitation is the potential publication bias. Studies with positive results may be more likely to be published, whereas those with null or negative findings may remain unpublished. This could lead to overestimation of the positive effects of probiotics. Future research should include unpublished studies or conduct a more thorough assessment of publication bias. Additionally, the sample sizes in some of the included studies were relatively small, which could have limited the statistical power to detect significant effects. Larger, well-powered studies are necessary to provide more robust evidence regarding the effect of probiotics on cognition and depressive symptoms in individuals with depression. Furthermore, while this meta-analysis focused on the effects of probiotics on cognitive function and depression, other relevant factors, such as dietary habits, lifestyle, and comorbid conditions, were not consistently controlled across all included studies. These uncontrolled variables could potentially confound observed outcomes. Additionally, our choice of the fixed-effects model, despite potential clinical heterogeneity arising from diagnostic tools, dosages, and intervention durations, may influence the interpretation of pooled results.

## 5. Conclusion

This study suggests that probiotics can positively influence cognitive domains and reduce depressive symptoms, indicating their potential adjunctive role in depression management. These findings underscore the intricate gut-brain axis relationship, with the gut microbiota likely influencing brain function and behavior. However, limitations such as variability in the methodologies of the included studies and short intervention durations highlight the need for larger, more standardized research to uncover the underlying mechanisms and confirm these preliminary insights.

## Author contributions

**Data curation:** Jiang He.

**Investigation:** Lemei Chang.

**Methodology:** Lemei Chang, Wenkai Wu.

**Resources:** Lange Zhang.

**Software:** Lange Zhang.

**Supervision:** Wenkai Wu, Dongyan Zhuo.

**Writing – original draft:** Jiang He.

**Writing – review & editing:** Dongyan Zhuo.
